# Review: Vaccine Myth-Buster – Cleaning Up With Prejudices and Dangerous Misinformation

**DOI:** 10.3389/fimmu.2021.663280

**Published:** 2021-06-10

**Authors:** Paul Löffler

**Affiliations:** Institute for Environmental Sciences, University of Koblenz-Landau, Landau, Germany

**Keywords:** immunization, vaccine safety, adjuvants, side effects, genetic vaccines

## Abstract

Although vaccines have already saved and will continue to save millions of lives, they are under attack. Vaccine safety is the main target of criticism. The rapid distribution of false information, or even conspiracy theories on the internet has tremendously favored vaccine hesitancy. The World Health Organization (WHO) named vaccine hesitancy one of the top ten threats to global health in 2019. Parents and patients have several concerns about vaccine safety, of which the ubiquitous anxieties include inactivating agents, adjuvants, preservatives, or new technologies such as genetic vaccines. In general, increasing doubts concerning side effects have been observed, which may lead to an increasing mistrust of scientific results and thus, the scientific method. Hence, this review targets five topics concerning vaccines and reviews current scientific publications in order to summarize the available information refuting conspiracy theories and myths about vaccination. The topics have been selected based on the author’s personal perception of the most frequently occurring safety controversies: the inactivation agent formaldehyde, the adjuvant aluminum, the preservative mercury, the mistakenly-drawn correlation between vaccines and autism and genetic vaccines. The scientific literature shows that vaccine safety is constantly studied. Furthermore, the literature does not support the allegations that vaccines may cause a serious threat to general human life. The author suggests that more researchers explaining their research ideas, methods and results publicly could strengthen the general confidence in science. In general, vaccines present one of the safest and most cost-effective medications and none of the targeted topics raised serious health concerns.

## Introduction

In times of impactful and threatening societal events or developments – such as climate change, economic or financial crises, terrorism, war or public health problems – many people make assumptions about the deceptiveness and evil intentions of powerful leaders or even entire branches (e.g. pharmaceutical industry, financial institutes, religions) due to the experience of substantial uncertainty and fear (1, 2). The belief in conspiracy theories (CTs) has been prevalent throughout human history (3–6).

As COVID-19 started spreading around the world, so did CTs about the virus, the evidence of sickness and even the vaccine, even before any vaccine had been registered, licensed or administered. This rapid distribution is tremendously favored by the internet. For example a Google search for immunization leads on the first page to several vaccine-critical sites and thus, might trigger the confirmation bias (7–9).

The first vaccine was introduced by Edward Jenner in 1796 and led to the worldwide eradication of smallpox (10, 11). Jenner extracted pus from a cowpox lesion on a milkmaid’s hand and inoculated an eight-year-old boy, which led to the boy’s immunization and, therefore, represents the basis of vaccine methodology (12, 13).

Immunization is widely recognized to be one of the greatest achievements for public health due to its success and cost-effectiveness (14). Vaccines have saved and continue to save millions of lives throughout the world (10). Thus, the World Health Organization has named vaccine hesitancy one of the top ten threats to global health in 2019 (15). Consequently, the anti-vaccine movement is having negative effects on individual and population health (16–19). In addition to the people directly protected by immunization, those unable to receive vaccines gain protection when a sufficient percentage (e.g., >80%) of the population is immunized. This “herd immunity” explains the ethics of solidarity regarding vaccination (20–22). Moreover, fully collaborative international effort and widespread vaccination can result in the decline and even eradication of persistent and serious diseases, as shown by the smallpox eradication in 1980 (23–27).

Presently, children receive most vaccines during their first years of life, as this is when they are most vulnerable to devastating infections. Such infections might be of invasive bacterial infection including pneumococcal or *Haemophilus influence* meningitis (10).

Even though vaccines are safer than ever before, the public perception has been affected by some severe incidents (28). For instance, during the first year following the vaccination campaign against the H1N1 infection in 2009 – 2010, the risk for narcolepsy increased up to 14-fold for children and adolescents and up to 7-fold for adults in several countries where the vaccine Pandemrix was used (Finland, France, Ireland, Norway, Sweden, the UK and the Netherlands) (29, 30). Though, an increased risk of narcolepsy after natural H1N1 infection was reported from China, where pandemic influenza vaccination was not used (31). Narcolepsy is a chronic sleep disorder characterized by excessive daytime sleepiness, which can have severe consequences for the patient. Two subtypes of narcolepsy have been described (narcolepsy type 1 NT1 and narcolepsy type 2 NT2), both of which have similar clinical profiles, except for the presence of cataplexy, which occurs only in patients with narcolepsy type 1 (32). HLA genes encoding the different antigen-presenting major histocompatibility complex (MHC) molecules have been associated with the development of NT1. The main genetic risk factor for narcolepsy is the HLA-DBQ1*06:02 allele (30, 31, 33–37). Depending on the population, up to 98 % of patients with NT1 carry the HLA-DBQ1*06:02 allele (33). Further, molecules that interact with MHC proteins such as T-cell receptors (TCR) have also been associated with the development of NT1 (32). A direct pathogenic link between narcolepsy and the vaccine has, however, remained elusive (30). Because narcolepsy appears to be dependent of a genetic predisposition, where responses to internal nucleoproteins seem to be a key trigger, vaccines containing only fragments of the pathogen such as genetic vaccines might constitute a safer approach, as they only present the spikes.

Limited safety data was available at the time of authorization of Pandemrix, since its development had been accelerated based on prior developments for other influenza viruses (38, 39). In total only 610 individuals were studied prior to authorization (39). This highlights a major change to the ongoing authorization procedure for COVID-19 vaccines, as no conclusion is drawn based on incomplete safety studies, rather a rolling review is implemented. Rolling reviews allow the European Medicines Agency (EMA) to assess data for promising medicines or vaccines as it becomes available instead of waiting until all trials have been concluded in order to start its work, during public health emergencies. Through these rolling reviews, EMA can start evaluating data while the development is still ongoing, and before the vaccine developer has submitted a request for marketing authorization (40). Further it shall be noted that the development of a vaccination concept against a new virus (e.g. SARS-CoV-2) might pose a greater challenge than the adaption of a well-established vaccine concept (e.g. influenza).

The objective of this work was to clear some of the prevalent myths by reviewing the current scientific literature. Therefore, five topics (formaldehyde, aluminium, mercury, autism, possible misconceptions regarding the COVID-19 vaccines) have been chosen based on the author’s perception of importance, as well as topicality, and elaborated in the following.

## Formaldehyde

Glenny and Hopkins accidentally discovered that formaldehyde can be used to detoxify several viral and bacterial toxins for vaccines, as they incubated the diphtheria toxin in vats previously cleaned with methanal (41, 42). The process of inactivation is a crucial step in vaccine production, as the inhibition of the replication of the virus is required, without reducing its antigenicity (43, 44). In the case of formaldehyde, the viral inactivation is achieved through the alkylation of amino and sulphydrilic groups of proteins and purine bases (45). Since its discovery, formaldehyde has had a long and extensive use in the formulation of both viral and bacterial vaccines. A comprehensive list of the formaldehyde detoxified vaccines (e.g., Havrix^®^ for Hepatitis A, Decavac™ and Adacel™ for Tetanus) can be found in the sixth book chapter by Finn and Egan (46, 47).

Recent studies indicate that excessive inactivation with formaldehyde causes unanticipated modifications to the respective antigen, which results in a reduced potency (48–51). This suggests that chemical inactivation might affect the protein conformation, leading to a loss of immunogenicity of the antigenic epitopes of a key surface protein, which is currently under discussion (49–58). Further, it was stated that the severity of chemical modifications depends on several factors such as incubation time, pH, temperature, formaldehyde concentration and ionic strength. Consequently, appropriate inactivation conditions during the vaccine production are essential in order to avoid unwanted changes of macromolecules (59–62).

Animal studies with birds found adverse effects of intramuscular formaldehyde-based vaccines such as reduced egg production, lowered estradiol and decreased antibody levels (63, 64). Formaldehyde was classified as carcinogen category 1B (reasonably suspected, primarily based on animal evidence) as well as mutagen category 2 (may induce heritable mutations in human germ cells) by the European Chemical Agency (ECHA) (65, 66). Furthermore, prolonged exposure *via* inhalation can cause nasopharyngeal cancer (adenomas) in rare cases and repeated contact with highly concentrated solutions can cause irritation, cell changes and squamous cell carcinoma (67).

Formaldehyde is ubiquitous in the environment (e.g., wood products, automobile fumes, paints, varnishes, carpets) and can be naturally derived from some food components (68–72). Smoking can even release up to 150 µg formaldehyde per cigarette (73–75). Additionally, recent research indicates that endogenously-produced formaldehyde contributes to the threat for human health (76, 77). Endogenous formaldehyde is generated by various essential mammalian metabolic processes, for example folate metabolism or histone, DNA and RNA demethylation reactions (75, 78–80). Thus, formaldehyde is omnipresent in human blood at an average concentration of 2-3 µg/mL (72). Consequently, mechanisms have evolved to counteract this genotoxic metabolite. The enzyme alcohol dehydrogenase 5 (ADH5) and the DNA-crosslink repair protein FANCD2 remove, as well as, mediate the damage of a formaldehyde detoxification (76).

The threshold level for formaldehyde in vaccines is 0.02 % (0.2 g/L) (81, 82). Additionally, nowadays the formaldehyde-based inactivation is followed by its removal. Thus, the amounts injected with vaccines are in a lower order of magnitude (max. 0.2 mg) than the metabolic *in situ* production (50 mg) and therefore regarded unproblematic by most scientists (72, 82, 83). A pharmacokinetic modeling study from 2013 assessing the safety of residual formaldehyde in infant vaccines also concluded that residual, exogenously applied formaldehyde continues to be safe following incidental exposures in infant children (84). Formaldehyde quantities in vaccines are accepted by regulatory authorities due to the high removal efficiencies after the inactivation. Further, the quantities are not additive to the amounts produced by the respective natural metabolism (72, 84).

## Aluminum

The use of aluminum (Al) adjuvants in vaccines has previously been investigated in 1926 by Glenny et al., who found that aluminum enhanced antigenicity in guinea pigs (85). Nowadays, many inactivated (or killed) vaccines such as diphtheria and tetanus toxoid would be less effective without aluminum salts [e.g., Al(OH)_3_, AlPO_4_, KAl(SO_4_)_2_ · 12 H_2_O (52, 86)]. The two common ways to prepare aluminum adjuvant vaccines are alum-precipitated and adsorbed vaccines. Adding a solution of aluminum salt to an antigen solution creates a precipitate of protein aluminate. The addition of the antigen to a preformed aluminum solution results in an aluminum-adsorbed vaccine (81, 87). It was demonstrated that not all aluminum adjuvants are equal either in terms of physical properties nor their biological reactivity and potential toxicity at injection site and beyond. For example, aluminum hydroxycarbonate adjuvants display a less pronounced extracellular uptake in comparison to clinically used aluminum hydroxide-based adjuvants (88).

The most relevant exposure to aluminum for the general population is by food. Aluminum in drinking water represents another, minor source of exposure (89–95). In general, the total dietary Al exposure of adults in the U.S. was calculated to be 7 – 9 mg/day in the 1990s and is stated as somewhat less nowadays (72, 91). Due to its cumulative nature in the organism after dietary exposure, the European Food Safety Authority (EFSA) decided on a tolerable weekly intake (TWI) for aluminum rather than a tolerable daily intake (TDI). Based on the combined evidence from toxicological studies, the EFSA established a TWI of 1 mg aluminum/kg body weight/week. This threshold value is assumed to be exceeded in many European countries due to the contamination of many cereals, cereal products, vegetables and beverages (89, 90, 96). The European Pharmacopoeia has set an aluminum threshold for vaccines at 1.25 mg per dose (82). This dosage is in accordance with the aforementioned European TWI of 1 mg aluminum/kg body weight/week. Moreover, vaccinations represent occasional instances rather than regular events.

The main carrier of aluminum ions in human plasma is the iron-binding protein transferrin, which enables the ions to enter the brain and reach the placenta and fetus (89, 97). The cellular Al-uptake is assumed to happen relatively slowly and most likely occurs from the aluminum bound to transferrin by transferrin-receptor mediated endocytosis (89). Most injected aluminum is excreted within two weeks *via* urine and feces (98–101). Another example describes elevated urinary Al was after repeated heroin use *via* inhalation from an aluminum foil (102). Al was shown to accumulate more in spleen, liver, bone and kidneys than in brain, other nervous tissues, muscle, heart or lung (90, 103, 104).

Although there have been allegations that aluminum adjuvants cause persistent myalgia, fatigue (105, 106) or autoimmune disease (107), no firm etiological association with vaccination has been established and the relationship between these conditions and aluminum adjuvants remains uncertain (108–111). Most allegations are based on a poor data situation and expert reviews have concluded that scientific evidence does not support them (72, 108). Despite the fact that no immediate hypersensitivity reaction could be monitored (108, 112–114), several case reports exist describing delayed hypersensitivity reactions (115–117), but so far no study had been able to find evidence for a link to aluminum (118). However, strong reactions with painful erythematous, pruritic eruptions, edema and blistering are rare (113, 119). Thus, more research is needed focusing on adjuvants to provide a safe alternative to Al-adjuvants for hypersensitive people.

In general, the U.S. Food and Drug Administration (FDA), as well as two scientific studies have concluded that episodic exposures to vaccines containing aluminum adjuvant continue to be an extremely low risk to infants, and that the benefits of using vaccines containing aluminum adjuvant outweigh any theoretical concern (120–122). As infants display the most vulnerable human stage, safety for the general population can be assumed as well.

## Mercury

After severe injuries and even deaths resulting from missing preservatives in faulty-produced vaccines in the 1920s, the newly found and investigated group of organomercury compounds sparked the hope to find safe vaccine preservatives (123). Thiomersal (or thimerosal), a white, crystalline powder, was one of the most promising organomercurials. Half of the weight was mercury in the form of ethylmercury bound to thiosalicylate (124). Consequently, the pharmaceutical company Eli Lilly & Co. patented the synthesis in 1926 (125).

As a preservative, thiomersal is to the bulk or final container added at the end of the production process, or it may be added to the diluent of a lyophilized vaccine (126). Further uses of thiomersal are in tattoo ink and products for contact lens care (127–129).

Following catastrophes in Minamata and Iraq, there was an increased focus on thiomersal, especially due to its similarity to ethylmercury and methylmercury (MeHg). In Minamata, Japan, methylmercury poisoning occurred in humans that ingested fish and shellfish contaminated by MeHg discharged in waste water from a chemical plant in 1956 (Chisso Co. Ltd.) (130). In 1971 and 1972, around 6530 farmers and family members in Iraq were hospitalized for methylmercury poisoning, of whom 459 died. The source was homemade bread out of seed wheat that had been treated with MeHg as fungicide (124, 131).

The U.S. FDA performed a risk assessment in 2001, which included calculations of maximum potential exposure to mercury from vaccines and determined that the cumulative mercury exposure from thiomersal of infants within their first six months may exceed the U.S. Environmental Protection Agency’s (EPA’s) reference dose (RfD) of 0.1 µg/kg/day (126, 132, 133). Although the effects of the thiomersal metabolite ethylmercury are understudied, most investigators based their risk assessment on studies of methylmercury, assuming similar toxicokinetics. Yet, Baker (2002) claimed that the chemical distinction is not trivial. He compared it to the different toxicity of ethanol (form of alcohol in beverages) and the highly lethal counterpart methanol, which differ only by one methylated sidechain in their structures (124). A study investigating the mercury levels in newborns and infants after receiving thiomersal-containing vaccines suggests the risk assessment should be conducted in light of the demonstrated short half-life of ethylmercury in newborns and infants after vaccination (134).

A thiomersal assessment by the American Academy of Pediatrics (AAP) in 2001 could not reveal evidence of harm caused by doses of thiomersal in vaccines, except for local hypersensitivity reactions. Nevertheless, the authors argue in support of the reduction and long-term removal of thiomersal from vaccines as a prophylactic precaution which would reinforce the public trust in immunization (126, 135). Hence, many manufacturers successfully removed thiomersal from their routine infant vaccines (124, 136). The European Medicines Agency (EMA) published a statement in 2004 with the same conclusion of missing toxicity for a mandatory removal, but argued for a voluntary reduction linked to the global goal of decreasing mercury exposure (137).

Studies showed that 0.01 % thiomersal is sufficient to sensitize children and, thus, could induce allergic responses, whereas the reason for the delayed hypersensitivity that occurs in 1 % of children is the thiosalicylic part (138, 139). In general, observed incidence of clinical symptoms related to thiomersal hypersensitivity is low (0.1%, 127, 140). Furthermore, Cox and Forsyth report thiomersal-sensitive people (based on contact studies) who declared that they received thiomersal-containing vaccines without complications (141). The risk of anaphylaxis from vaccines was estimated to be 1.31 (95 % confidence interval) per million vaccine doses and is consequently considered low (142). A list of vaccines containing thiomersal, provided by the Johns Hopkins University, can be found online at: www.vaccinesafety.edu (143).

## Vaccines Cause Autism

The hypothesized link between the measles, mumps, rubella (MMR) vaccine and autism has challenged vaccine acceptance for the past 22 years, following a later-retracted *Lancet* publication from 1998. A. J. Wakefield et al. (144) published a case series study investigating unexpected intestinal lesion in twelve children. With eight of these children, the author found a new variant of autism characterized by gastrointestinal disorder and developmental regression, which he linked to the MMR vaccine (144). Wakefield hypothesized that the measles virus had triggered inflammatory lesions in the colon, disrupting the permeability of the colon through which neurotoxic proteins reach the bloodstream and the brain, thus causing autism. Investigations revealed that Wakefield received money from an attorney’s office, which also showed connections to the children of Wakefield’s study (145, 146). Consequently, ten of the twelve co-authors published a retraction of Wakefield’s interpretation and declared that in the publication no causal link was established between MMR vaccine and autism as the data was insufficient (147). Likewise, the journal retracted the publication and Wakefield was barred from practicing medicine (148–150). Numerous other studies found no significant association between the MMR vaccine or the mumps virus and autism spectrum disorder (ASD) (72, 81, 151–164). A recent nationwide cohort study in Denmark by Hviid et al. (164) used the Danish population registries to evaluate whether the MMR vaccine increased the risk for autism in children, subgroups of children or time periods after vaccination. Using the data of more than 650 000 children born in Denmark between 1999 and 2010 no increased risk for autism or triggering autism in susceptible children could be determined. This supports prior findings with significant additional statistical power (164). One study found higher mercury concentrations in the blood of autistic children which was not related to vaccines. Therefore, they link the environmental pollution of mercury and lead to the development of autism (165). A systematic review found that studies with the lowest bias based on study quality criteria did not support a causal association between the MMR vaccine and ASD (156, 163). Nevertheless, the molecular mechanisms that underlie ASDs are not yet known. Thus, epidemiological studies provide the statistical tool to exclude a correlation between ASD and vaccines so far. So far, a strong and complex genetic component, with multiple familial inheritance patterns and an estimate of up to 1000 genes potentially involved, is assumed to contribute to the development of ADS (166–168). Many vaccines are administered to 12- to 18-month-old children, which coincides with the age of the first signs of an impending development condition, such as ADS. Thus, the difference between temporal correlation and causal relationship of events might not be recognized (169).

This persistence of information proven to be false in the public memory highlights the importance of scientific accuracy in research as well as the caution in premature interpretation, as there is no evidence for an association between vaccines and autism (170, 171).

## Review of Possible Misconceptions Regarding COVID-19 Vaccines

The severe acute respiratory syndrome coronavirus type 2 (SARS-CoV-2) infection and the resulting coronavirus disease 2019 (COVID-19) are an international public health emergency with devastating health consequences as well as major socio-economic disruptions. Thus, safe and effective vaccines are urgently needed. Some of the candidates and the first to be approved were mRNA vaccines, which appears to be a rather new concept of vaccination in the public eye. In general, the concept of genetic (DNA & RNA) vaccines was raised and first investigated several decades ago with the hope of easy-to-produce, safe and effective vaccines (172, 173). In comparison to virus-based vaccines, messenger RNA (mRNA)-based vaccines present additional safety features (174). In general mRNA vaccines carry transcripts encoding antigens, and use the translational machinery in the recipient’s cell to produce antigens, which then stimulate an immune response (175, 176).

Due to the wide media attention of the registration of the first mRNA-based vaccines in Europe, justified concerns regarding the technology developed fast into misbeliefs vastly spreading *via* social media. For example, one of the main fears describes the alteration of the recipient’s genome *via* the injected RNA (177–180). But, because mRNA is sensitive to the omnipresent ribonucleases (RNase) and its metabolic decay occurs within a few days, the risk of genomic integration is considerably lower when compared to DNA-based vaccines (181–183). Moreover, there is little chance of mRNA interaction with the genome because mRNA does not enter the nucleus. Most studies investigating the potential DNA integration into the host cell genome, found either no integration or levels, that were several orders of magnitude below the spontaneous mutation frequency and thus, were not considered to pose a significant safety concern (184–186). Nevertheless, recombination between single-stranded RNA molecules may occur in rare cases and could engender crossing-over events, as well as decrease the immunization efficacy (183, 187–190). While the entry of DNA vaccines into the nucleus brings technical challenges, it also carries the risk of insertional mutagenesis, which might disrupt gene functions or promote oncogenic development (176, 183, 191, 192).

Better scientific communication of the current state of research on genetic vaccines could reduce the impression of an experimental method, what might result in a reduced vaccine hesitancy (177). In 1999 for example, genetic vaccines entered clinical trials testing safety and efficacy in healthy human volunteers (185). In 2018 the U.S. FDA and the EMA approved the first RNA based drug called Onpattro (patisiran). The injected drug treats patients with polyneuropathy (peripheral nerve damage) caused by hereditary transthyretin amyloidosis (hTTR), which is a genetic disease caused by the build-up of an abnormal protein in the nerves, heart, and/or gastrointestinal tract (193–196). Many studies investigating phase I/II clinical studies of mRNA vaccines provide promising results regarding antitumor treatment approaches (197–201).

Another issue of DNA vaccines, that might rise concerns, are autoantibodies. Autoantibodies are specific for self-antigens and can cause damage to cells and tissues and result in autoimmune diseases such as systemic lupus erythematosus. The fear of adverse side effects or such long-term complications depicts another factor for vaccine hesitancy. In comparison to DNA-based vaccines, no mechanism is known for mRNA-based vaccines to induce pathogenic anti-DNA autoantibodies (202, 203). DNA vaccines are mostly composed of an antigen-encoding gene on a plasmid backbone of bacterial DNA. Because the plasmid backbone is of bacterial origin, it might have immunomodulatory properties that can cause the production of autoantibodies as the immune system identifies it as foreign to the body (204, 205). As mRNA provides the minimal genetic construct, it harbors only the elements directly required for expression of the encoded protein (183). Thus, the risk of autoantibody formation is minimized.

Besides the safety benefits, mRNA is easy to produce and purify (174). As most viral vaccines are produced by cultivating the virus using e.g., fertilized bird eggs or other animal cells, the use of mRNA would simplify the production process (206, 207).

In December 2020, the first COVID-19 vaccine was approved by the EMA and all respective research data published (198–200). The vaccine called BNT162b2 or Comirnaty (CAS: 2417899-77-3) encodes a P2 mutant spike protein (PS-2), produced by the cooperation of the pharmaceutical companies BioNTech and Pfizer. It is a two-dose lipid nanoparticle-formulated nucleoside-modified mRNA vaccine and was placebo-controlled and observer-blinded investigated amongst more than 40,000 participants. There were eight cases of COVID-19 with onset at least 7 days after the second vaccination dose among participants assigned to receive BNT162b2 and 162 cases among those assigned to the placebo. This illustrates an efficacy of 95 % (95 % confidence interval). Even across subgroups defined by age, sex, race, ethnicity, baseline body-mass index, and the presence of coexisting conditions, similar efficacies were observed. The safety profile of BNT162b2 was characterized by occasional short-term, mild-to-moderate pain at the injection site, fatigue and headache (199–201, 208–210). Cabanillas et al., (211) raised concerns regarding hypersensitivity to the adjuvant polyethylenglycol (PEG) (211). PEG forms a protective hydrophilic layer, sterically stabilizing the lipid nanoparticles and, thus, contributes to the storage stability of the vaccine (212). Because immediate PEG hypersensitivity may be underestimated, an immediate reaction test on the skin might be of advantage to prevent adverse reactions (213, 214). PEG exemplifies a hydrophilic polymer which is an authorized food additive (E 1521) with a maximum limitation of 10 g PEG per kg food in the European Union (215, 216). Although anaphylactic reactions to PEG have been reported with increasing frequency over recent years, its mechanism is still unknown and the allergenic potential often overlooked (211, 217). Recent publications advise patients with known allergies to vaccine components to consult allergists before vaccination (218, 219). Generally, immediate life-threatening reactions are very rare, as 1.3 cases per million doses are reported (220).

Another promising vaccine candidate for the prevention of SARS-CoV-2 is mRNA-1273 by the pharmaceutical company Moderna, which encodes the stabilized prefusion SARS-CoV-2 spike protein (S-2P) (221–224). The EMA recommended the vaccine for authorization at the beginning of January 2021 (225). The clinical trial involving more than 30000 people showed an efficacy of 94.1 % reduction in the number of symptomatic COVID-19 cases. “The trial also showed a 90.0 % efficacy in participants at risk of severe COVID-19, including those with chronic lung disease, heart disease, obesity, liver disease, diabetes or HIV infection. The high efficacy was also maintained across genders, racial and ethnic groups” (226).

Nevertheless, more vaccine candidates are needed to grant an equal immunization without vaccine nationalism. Therefore, the COVAX Facility has been established, which is an international partnership that aims to financially support leading vaccine candidates and ensure access to vaccines for lower-income countries (227). In general, genetic vaccines display promising future candidates for several diseases as they are fast and easy to produce whilst harboring a comparably low risk. Especially, mRNA-based vaccines pose a low risk, as they are unlikely to interact with the human genome and the risk for autoantibody-formation leading to autoimmune diseases is minimized.

## Conclusion

In this article, several common vaccine safety controversies are summarized, and the current literature reviewed. Since all topics and references were selected based on the author’s perception of importance bias cannot be excluded, what poses a clear limitation of the article. However, this article was unable to identify an alarming health threat, mostly because threshold values by risk assessments gave no cause for concerns. Further possible misconceptions of COVID-19 vaccines were highlighted and assessed to be mostly harmless. However, the vastly spreading misinformation concerning vaccine safety poses a threat especially to children’s lives worldwide. Palamenghi et al. (228) correlated the willingness to vaccination with the COVID-19 vaccine to the general trust in research and assessed that the proportion of citizens that intend to get the COVID-19 vaccine is probably too small to effectively stop the spreading of the disease (228). Therefore, the noted deficits regarding scientific communication are of high concern. Most publications are not easy to understand, especially for people without scientific knowledge. Thus, more scientists should publicly report their research ideas, methods and results in a balanced manner, which could strengthen the general public confidence in science (229, 230). Furthermore, many results are hidden behind a paywall that is often costly, which, therefore, is another barrier for the accessibility of scientific publications.

## Author Contributions

The author confirms being the sole contributor of this work and has approved it for publication.

## Funding

The publication was funded by the Open Access Fund of the University of Koblenz-Landau.

## Conflict of Interest

The author declares that the research was conducted in the absence of any commercial or financial relationships that could be construed as a potential conflict of interest.
